# Determinant factors for adherence to antiretroviral therapy among adult HIV patients at Dessie Referral Hospital, South Wollo, Northeast Ethiopia: a case–control study

**DOI:** 10.1186/s12981-021-00365-9

**Published:** 2021-07-13

**Authors:** Mehd Abdu, Betelhem Walelgn

**Affiliations:** 1 Department of Nursing, College of Medicine and Health Science, Mizan-Tepi University, Mizan Teferi, Ethiopia; 2 Department of Comprehensive Nursing, School of Nursing, College of Health Science, Woldia University, Woldia, Ethiopia

**Keywords:** Adherence, Antiretroviral therapy, Dessie Referral Hospital, HIV/AIDS

## Abstract

**Introduction:**

World health organization defined adherence as the extent to which a person’s behavior – taking medications, following a diet, or executing lifestyle changes correspond with agreed recommendations from the health care provider. There is a contradiction among studies and previous studies conducted in the study area used a cross-sectional study design. This study aimed to identify determinant factors for adherence to antiretroviral treatment among people living with HIV at Dessie Referral Hospital by using an unmatched case–control study design.

**Methods and materials:**

an institution-based unmatched case–control study design was used on a total sample of 582 (146 controls and 436 cases). Each respondent was selected by consecutive random sampling. The collected data were entered and analyzed by using Statistical Package for Social Science version 25.0. Multivariable binary logistic regression analysis was used to identify variables that were statistically significant determinants.

**Result:**

The mean age of the respondents was 41.64 years. About 61.5% of the participants were females. Patients with baseline HIV stage I was more likely to be adherent to the prescribed HIV medicine (AOR: 2.194 95% CI: 1.116, 4.314) as compared with those with baseline WHO stage IV. Patients who did not take anti-tuberculosis medication collaterally with the prescribed HIV medicine were more likely to be adherent (AOR: 2.271 95% CI: 1.257, 4.102). Patients who took antiretroviral therapy for more than 24 months were more likely to be adherent (AOR: 3.665 95% CI: 1.321, 10.170).

**Conclusion:**

Initiation of antiretroviral therapy at the later stage of the disease and taking anti-tuberculosis concomitantly were negatively associated with adherence. Being on antiretroviral therapy for a longer duration has a positive association. Health facilities and professionals should strictly apply strategies for the prevention of tuberculosis among HIV patients to avoid concomitant use of anti-tuberculosis medications.

## Introduction

Human immunodeficiency virus has a great role in the ill-health of humans worldwide. At the end of 2019, about 38.0 million people were living with HIV worldwide and 1.7 million people became newly infected. It claimed about 33 million lives so far. The larger proportion of the epidemic (25.7 million people living with HIV) was in the WHO Africa region [1]. In Ethiopia, about 613,000 people lived with HIV; among these, 62 percent were females [2].

Antiretroviral therapy (ART) is the act of treating a patient with the medications of HIV [3]. Screening for and management of different opportunistic infections ahead of starting ART is an important aspect of care. Once the diagnosis of HIV infection is confirmed, immediate induction of antiretroviral treatment is recommended regardless of the CD_4_ count and WHO HIV stage of the patient [4]. Patients who are co-infected with tuberculosis should start ART within fourteen days of initiation of anti-TB [5].

World health organization defined adherence as the extent to which a person’s behavior–taking medications, following a diet, and/or executing lifestyle changes corresponds with agreed recommendations from the health care provider. An extremely high level of continuous adherence to ART is very important to decrease viral replication and for better immunological and clinical outcomes [4].

Non-adherence to the prescribed antiretroviral treatment is a major challenge in the management plan of HIV. A study done at Dessie Referral Hospital showed that about 82.3% of HIV patients on ART were adherent to their HIV medications. Another study conducted in southern Ethiopia showed that 77.10% of the respondents were adherent to the prescribed antiretroviral therapy [6, 7].

According to different studies, residence, educational level, occupation, co-morbid conditions, disclosure of HIV status, CD_4_ cell count, substance use, and social support were identified as risk factors to be associated with the adherence status of PLWHIV for their ART [8–10]. HIV patients who are not adherent to their HIV medicine are prone to several adverse outcomes. Non-adherence was identified as a major risk factor for the development of ART treatment failure [11]. Sub-optimal adherence results in a higher risk of transmitting HIV, developing drug resistance, the rapid advancement of the disease, and death [4, 5].

World health organization and national guidelines recommended using each facility visit as an opportunity for assessing and supporting treatment adherence among people living with HIV. Viral load monitoring is considered a gold standard for assessing treatment adherence for HIV patients [4, 5].

There is a contradiction among studies conducted regarding factors determining adherence to ART. Conducted studies in the selected area used a cross-sectional study design. Therefore, the aim of conducting this study was to identify determinant factors for adherence to antiretroviral treatment among HIV patients having follow-up at ART clinic of Dessie Referral Hospital with an unmatched case–control study design.

## Methods and materials

### Study area

South Wollo zone in Amhara region is bordered by North Shewa and Oromia region on the south, by East Gojjam on the west, by South Gondar on the Northwest, by north Wollo on the North, by Afar region on the northeast, and by Oromia Zone on the east. There are 20 Woredas in the zone. The administrative center of south Wollo, Dessie, is located 401 km away from the capital city of Ethiopia, Addis Ababa. Dessie referral hospital, the only referral hospital in south Wollo, is providing several services for the societies and adjacent communities outside the South Wollo zone. These services include outpatient services, inpatient services, emergency care, obstetric and gynecologic services, cancer screening and treatment, surgery, maternal and child health, and ART service. 6350 patients had a follow-up at the ART clinic of Dessie Referral hospital. Among these, 4831 were adults at the age of 18 years old and above.

### Study design and period

This study used an unmatched case–control study design to identify determinant factors for patient’s adherence to ART among patients living with HIV/AIDS. The study was conducted in March 2020.

### Source and study population

#### Source population

Cases: All adult patients on ART at Dessie Referral Hospital who were adherent to their HIV medications.

Controls: all adult patients on ART at Dessie Referral Hospital who were poorly adherent to the prescribed HIV medicine.

#### Study population

Cases: Randomly selected adherent adult HIV patients who were available during the data collection period.

Controls: Randomly selected poorly adherent adult HIV patients who were available during the data collection period.

### Eligibility criteria

HIV patients with the age of  ≥ 18 years and on ART for at least six months were included in the study. Those patients who came to start ART during the data collection period and those lost to follow-up patients were excluded.

#### Sample size determination and sampling technique

The sample size for this study was calculated by double population proportion formula by using EpiInfo version 7.2.3.1 software with the consideration of 95% Confidence level, power of 80%, and control to case ratio of 1:3. The sample size was computed for each independent variable which was identified as a statistically significant determinant factor by the selected study. Then, the variable which was found to result in a higher sample size (CD_4_ count) was used. The calculated higher sample size was 554. The final sample size became 582 by adding a non-response rate of 5% (146controlsand436cases) [9]. Individual respondents were selected by using a consecutive random sampling technique.

#### Data collection instrument and procedures

Structured interviewer-administered questionnaires and reviewing the medical record of the patient were used to collect the data for this study. Clinical data necessary for this study were obtained from patient cards. Other information (socio-demographic characteristics and host/personal characteristics) was gathered by interviewing the patient using a structured questionnaire. First, the patient was interviewed and then after the patient exited, other data from the medical record was taken. The questionnaires were adopted after careful reviewing of different works of literature. Experts reviewed the questionnaires to check them for face validity. Adherence was assessed by a dose adherence method used by Ethiopian Federal Ministry of Health in which the percentage of doses the patient never missed in the past 30 days was calculated. This was done by number of doses taken (prescribed doses minus missed doses) divided by the number of prescribed doses and multiplied by 100 [6].

### Study variables

Dependent variable: Adherence to ART.

Independent variables: Sociodemographic characteristics—Age, gender, marital status, residence, educational status, occupation, monthly income, family size. Host/personal factors—Substance use (alcohol, smoking, khat), functional status. Clinical/immunological factors—opportunistic infections, DM, baseline WHO stage of HIV, baseline CD_4_ cell count, IPT use, BMI, duration on ART, viral load, taking anti-TB drugs.

### Operational definitions

Khat: a flowering plant having an evergreen leaves which contains cathinone, a stimulant which causes excitement, loss of appetite, and euphoria.

Adherence: The patient with a percentage of ≥ 95% by dose adherence scale used by Ethiopian Federal Ministry of Health was considered as adherent, and the others was considered as non-adherent [6].

Functional status: Working – if the patient can perform his/her habitual works in or out of the house; Ambulatory – if the patient can carry out activities of daily living (ADLs); Bedridden—the patient cannot able to carry out activities of daily living (ADLs) [12].

### Data quality assurance

A pretest was done on 5% of the calculated sample size to check for the consistency of the questionnaires. First, the English version questionnaires were prepared, translated to the Amharic version, and then back-translated to the English version to check for consistency. 4 BSc nurses who have had experience at the antiretroviral therapy clinic collected the data. An MSc public health professional supervised the data collectors. Data collectors took a two-day training. The data collectors received detailed and comprehensive instructions from the principal investigator to assure the quality of the data. The filled questionnaire was checked for completeness.

### Data processing and analysis

After confirmation of completeness and consistency of the collected data, the questionnaire was coded, entered into, and analyzed by SPSS version 25.0. Hosmer–Lemeshow goodness of fit test was checked for the entered data to evaluate the fulfillment of the assumptions for binary logistic regression and to evaluate model fitness. For each independent variable with dependent variables, bivariable binary logistic regression was done. Covariates with a p-value of ≤ 0.25 in a bivariable binary logistic regression analysis were considered eligible for multivariable binary logistic regression analysis [12]. A variable with a P-value of ≤ 0.05 in the multivariable binary logistic regression analysis was assumed to be a statistically significant determinant factor for adherence to ART. The findings of the study were described and expressed by using tables, graphs, and narrative descriptions.

## Result

### Socio-demographic data of respondents

Of 582 selected individuals, 556 were participated (417 cases and 139 controls) . The response rate for all participants became 95.5%. The response rate of cases was 95.6% and of controls was 95.2%. The mean age of the study participants was 41.64 years. About 61.5% of the participants were females. More than half, (59.3%), of the participants were married. More than half, 319(57.4%), of the respondents were Muslim. More than one quarter (28.1%) of the respondents were uneducated. More than one-third (39.7%) of the respondents were self-employed. The mean monthly income of the respondents was 2141.02 Ethiopian birr. More than half, 330(59.4%) of the respondents reside in urban areas (Table 1).

**Table 1:** Sociodemographic status of HIV patients at DRH, 2020. (N = 556)

Variables	Categories	Cases		Controls		Total	
		N	%	N	%	N	%
Age (mean ± SD)		41.40 ± 11.742		42.37 ± 11.589		41.64 ± 11.701	
Gender	Male	165	39.6	49	35.3	214	38.5
	Female	252	60.4	90	64.7	342	61.5
Marital status	Single	51	12.2	20	14.4	71	12.8
	Married	242	58.0	88	63.3	330	59.3
	Divorced	61	14.6	15	10.8	76	13.7
	Widowed	63	15.1	16	11.5	79	14.2
Religion	Orthodox	149	35.7	56	40.3	205	36.9
	Muslim	244	58.5	75	54.0	319	57.4
	Protestant	22	5.3	6	4.3	28	5.0
	Others	2	0.5	2	1.4	4	0.7
Educational status	Uneducated	127	30.5	29	20.9	156	28.0
	Primary school	113	27.1	36	25.9	149	26.8
	Secondary school	109	26.1	42	30.2	151	27.2
	Tertiary education	68	16.3	32	23.0	100	18.0
Occupation	Self-employed	170	40.8	51	36.7	221	39.7
	Gov’t employed	57	13.7	19	13.7	76	13.7
	Unemployed	22	5.3	10	7.2	32	5.8
	Housewife	70	16.8	27	19.4	97	17.4
	Farmer	77	18.5	25	18.0	102	18.3
	Student	21	5.0	7	5.0	28	5.0
Family size	1	14	3.4	2	1.4	16	2.9
	> 2	403	96.6	137	98.6	540	97.1
Residence	Urban	248	59.5	82	59.0	330	59.4
	Rural	169	40.5	57	41.0	226	40.6

*gov’t* *Government*

### Host/personal characteristics of study participants

**Fig. 1:**
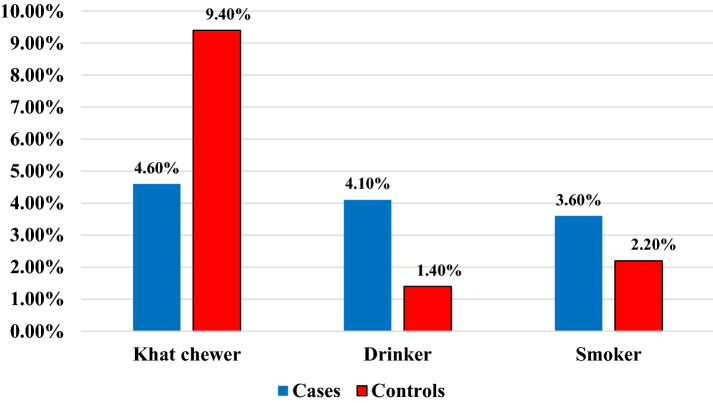
Host and personal characteristics of HIV patients at DRH, 2020

**Fig. 2:**
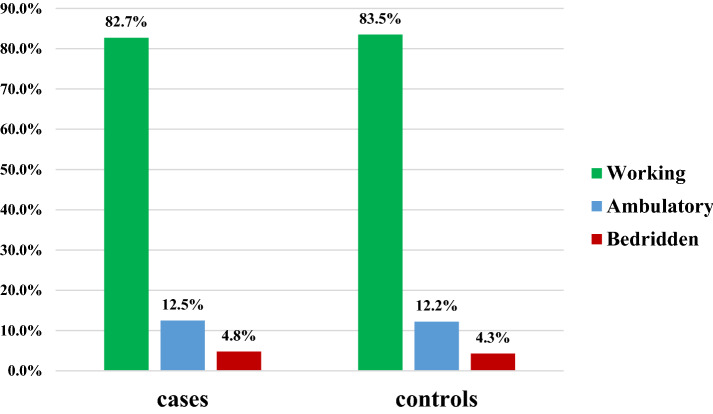
Functional status of HIV patients at DRH, 2020

The higher amount of cases, 398(95.4%), and controls, 126(90.6%), did not chew khat. The higher proportion of cases, 400(95.9%), and controls, 137(98.6%), were not alcohol drinkers. The majority of cases, 402(96.4%), and controls, 136(97.8%), did not smoke a cigarette (Fig. 1). The majority of cases, 345(82.7%), and controls, 116(83.5%), had working functional status (Fig. 2).

### Clinical/immunological status of the respondents

The higher proportion of the participants, 521(93.7%), had no history of DM. The majority of cases, 343(82.3%), and controls, 109(78.4%), had no history of opportunistic infections. The respondents had a mean follow-up of 94.63 months with an SD of 39.124. While starting ART, 170(40.8%) of cases were in WHO stage I. However, a higher percentage of controls, 55(39.6%), start ART at the second stage of the disease (stage II). The respondents have had a mean baseline CD4 count of 577.08 cells/mm_3_ with an SD of 322.91. The majority of cases, 291(69.8%), and controls, 92(66.2%), had a baseline viral load of < 1000 copies/ml (Table 2).

**Table 2:** Clinical/immunological status of HIV patients at DRH, 2020. (N = 556)

Variables	Categories	Cases		Controls		Total	
		N	%	N	%	N	%
Diabetes mellitus	Yes	27	6.5	8	5.8	35	6.3
	No	390	93.5	131	94.2	521	93.7
Having OIs	Yes	74	17.7	30	21.6	104	18.7
	No	343	82.3	109	78.4	452	81.3
On ART (mean ± SD)^a^		96.77 ± 38.909		88.21 ± 39.209		94.63 ± 39.124	
Baseline HIV stage	Stage I	170	40.8	40	28.8	210	37.8
	Stage II	122	29.3	55	39.6	177	31.8
	Stage III	89	21.3	23	16.5	112	20.1
	Stage IV	36	8.6	21	15.1	57	10.3
CD_4_ count(mean ± SD)^b^		567.11 ± 317.016		606.93 ± 339.39		577.08 ± 322.910	
BMI (mean ± SD)^c^		18.821 ± 1.9556		18.797 ± 1.8771		18.815 ± 1.9347	
IPT use	Yes	304	72.9	83	59.7	387	69.6
	No	113	27.1	56	40.3	169	30.4
Taking anti-TB drugs	Yes	33	7.9	26	18.7	59	10.6
	No	384	92.1	113	81.3	497	89.4

^*a*^*in month*

^*b*^*in cells/mm*^*3*^

^*c*^*in kg/m*^*2*^

### Factors associated with adherence to ART

Explanatory variables with a p-value of < or = 0.25 in the bivariable binary logistic regression analysis were used for the computation in the multivariable logistic regression analysis.

These explanatory variables were marital status, educational level, monthly income, Khat chewing, alcohol drinking, duration of taking ART, baseline WHO stage, IPT use, and being on the full course of anti-tuberculosis medications. To identify determinants that are independently associated with adherence to ART, multivariable logistic regression analysis was used. Three variables became an independent determinant for adherence to ART after adjusting the effect of other variables for the outcome.

Baseline HIV stage was a statistically determinant factor for adherence to ART in which patients with baseline HIV stage I was more likely to be adherent to the prescribed HIV medicine (AOR: 2.194 95% CI: 1.116, 4.314) as compared with those patients with baseline WHO stage IV. Collateral use of the full course of anti-tuberculosis drugs was a statistically determinant factor for adherence to ART in which patients who did not take anti-TB medication were more likely to be adherent to antiretroviral therapy (AOR: 2.271 95% CI: 1.257, 4.102) as compared with those who took anti-TB medication collaterally with the prescribed HIV medicine.

The duration in which the patient was on ART was a statistically determinant factor for adherence to ART in which patients who took ART for more than 24 months were more likely to be adherent to the prescribed HIV medicine (AOR: 3.665 95% CI: 1.321, 10.170) as compared with those patients who were on ART for 12 months or less (Table 3).

**Table 3:** Factors determining adherence to ART among HIV patients at DRH, 2020

Variables	Categories	Case	Control	COR (95% CI)	p-value	AOR (95% CI)	p-value
Marital status	Single	51	20	1			
	Married	242	88	1.078 (0.609, 1.910)	.796		
	Divorced	61	15	1.595 (0.741, 3.430)	.232		
	Widowed	63	16	1.544 (0.727, 3.282)	.259		
Educational status	Uneducated	127	29	1			
	1o education	113	36	0.717 (0.413, 1.243)	.236		
	2o education	109	42	0.593 (0.346, 1.015)	.057		
	3o education	68	32	0.485 (0.271, 0.869)	.015		
Monthly income	< = 2000 birr	273	99	1			
	> 2000 birr	144	40	1.305 (0.859, 1.985)	.212		
Khat	Yes	19	13	1			
	No	398	126	2.161 (1.038, 4.500)	.039		
Alcohol	Yes	17	2	1			
	No	400	137	0.343 (0.078, 1.506)	.156		
Duration on ART	6–12 month	8	9	1		1	
	13-24 month	9	2	5.062 (0.833, 30.75)	.078	4.835 (0.767, 30.504)	.094
	> 25 month	400	128	3.516 (1.329, 9.301)	.011	3.665 (1.321, 10.170)	0.013^*^
Baseline WHO stage	Stage I	170	40	2.479 (1.309, 4.697)	.005	2.194 (1.116, 4.314)	.023^*^
	Stage II	122	55	1.294 (0.692, 2.418)	.419	1.065 (0.554, 2.045)	.851
	Stage III	89	23	2.257 (1.113, 4.577)	.024	1.991 (0.958, 4.136)	.065
	Stage IV	36	21	1		1	
IPT use	Yes	304	83	1.815 (1.214, 2.713)	.004		
	No	113	56	1	1		
Taking anti-TB	No	384	113	2.677 (1.537, 4.664)	.001	2.271 (1.257, 4.102)	.007^*^
	Yes	33	26	1		1	

^*^Statistically significant (p-value ≤ 0.05)

## Discussion

According to the study, some variables were identified as statistically significant determinant factors for adherence to ART. These factors include baseline HIV WHO stage of the patient, collateral use of the full course of anti-TB medications, and the duration in which the patient was on ART.

Based on this study, baseline HIV stage is statistically associated significantly with adherence to ART in which patients with baseline HIV stage I was more likely to be adherent (AOR: 2.194 95% CI: 1.116, 4.314) as compared with patients with baseline HIV stage IV. This is similar to a retrospective study done in northern Ethiopia in which those patients with HIV stage II(AHR: 0.47 95% CI: 0.36, 0.60), WHO stage III (AHR: 0.25 95% CI: 0.19, 0.34), and WHO stage IV (AHR: 0.57 95% CI: 0.41, 0.81) had a lower risk to be adherent to the prescribed HIV medicine as compared with HIV stage I [13]. This may be due to the effect of serious opportunistic infections developed in the advanced stage of the disease. HIV selectively affects activated white blood cells. These white blood cells are essential for the functionality of the antigen-specific immune response of the body [14]. During the advanced stage of the disease, patients develop different local and systemic infections secondary to compromised immune function. With such clinical syndromes, the patient may not be able to tolerate the side effects of the HIV medicine and more likely to miss taking and be non-adherent to it [6]. Ethiopian national strategies recommended the use of chemoprophylaxis (co-trimoxazole) and starting ART in the early stage of the disease for the prevention of the occurrence of opportunistic infections [15]. Ethiopian guideline for comprehensive HIV prevention, care, and treatment supports that induction of antiretroviral treatment in the early HIV stage has an important role in better clinical outcomes and improved survival of the patient [4].

According to the study, being on the full course of anti-tuberculosis medications is statistically associated significantly with patients’ adherence to the prescribed HIV medicine in which HIV patients who did not take the full course of anti-TB were more likely to be adherent to antiretroviral therapy (AOR: 2.271 95% CI: 1.257, 4.102) as compared with those who did not take anti-TB medicine. A cross-sectional study done in Debre-Birhan Referral Hospital and health center also found that patients who took ART while taking traditional, complementary, and alternative medicine were less likely to be adherent to the prescribed ART (AOR 4.7 95% CI 1.06, 21.22) [16]. Similarly, a study conducted at Dessie referral hospital showed that patients who took medications other than ARV were less likely to be adherent to the prescribed HIV medicine [7]. This finding is also similar to an international trial in which patients who were taking a concomitant drug were less likely to be adherent to the prescribed ART (AOR: 0.82 95% CI: 0.75, 0.89) [17]. This may be due to the increased pill burden to the patient. As the patient takes different medications, there is an increased chance of developing too many side-effects and drug interactions [4]. These conditions may create unpleasant feelings for the patient. Hence, the patient may miss taking the medication and becomes non-adherent to it.

This study found that the duration in which the patient was on ART is statistically associated significantly with adherence to ART. Patients who took ART for more than 24 months were more likely to be adherent (AOR: 3.665 95% CI: 1.321, 10.170) as compared with those who took ART for 12 months or less. Other studies also supported that patients would be non-adherent at the early period of infection in which a study done in Nepal found that patients on antiretroviral therapy for more than three years were more likely to be adherent to ART (AOR: 10.055 95% CI: 2.383, 42.430) as compared with those on HIV for less than three years [18]. This may be due to increased risk of IRIS (Immune Reconstitution Inflammatory Syndrome) and occurrence of other opportunistic infections in the early months of taking ART. During the early months of starting ART, viral suppression and immunological improvement are not expected and the patient is less likely to be adherent [5]. In contrast, a study conducted in northern Ethiopia showed that patients who were on ART for more than two years were more likely to be non-adherent (AOR: 7 95% CI: 2.2, 22.6) as compared with those who were on ART for 6–12 months [19]. This difference may be due to a variation in the sample size. The number of participants involved in the study has a crucial effect on the representativeness of samples for the general population and the precision of the collected data. A study conducted with a larger number of participants is more representative of the whole population; whereas, a study conducted on a smaller number of participants tends to result in less accurate estimation and a higher rate of sampling error (20).

### Limitation of the study

Questions related to substance use are predisposed to social desirability and recall bias which may influence the accuracy of the information. The accuracy of the information may be affected by using medical records as a source of information.

## Conclusion

This study found some factors as a determinant factor for adherence to the prescribed antiretroviral treatment. These factors include baseline HIV WHO stage, being on the combined anti-tuberculosis drugs, and the duration in which the patient was on ART. Induction of antiretroviral treatment at the advanced stage of HIV (stage IV) and being on the combined anti-tuberculosis medications were associated negatively with adherence to ART. Being on ART for a longer duration has a positive association with adherence to prescribed HIV medicine. Health facilities and professionals should strictly apply strategies for the prevention of tuberculosis among HIV patients to avoid concomitant use of anti-TB medications. Health institutions and health care providers should provide a strict and intensive adherence support for patients with advanced stage of HIV, for patients taking anti-TB drugs, and during the early days of treatment.

## Data Availability

The datasets used and/or analyzed during the current study are available from the corresponding author on reasonable request.

## Abbreviations

AIDS: Acquired immunodeficiency syndrome
AHR: Adjusted hazard ratio
AOR: Adjusted odds ratio
ART: Antiretroviral therapy
BMI: Body mass index
CI: Confidence interval
COR: Crude odds ratio
DM: Diabetes mellitus
DRH: Dessie Referral Hospital
HIV: Human immuno-deficiency syndrome
IPT: Isoniazid preventive therapy
IRIS: Immune reconstitution inflammatory syndrome
OI: Opportunistic infection
PLWHIV: People living with human immuno-deficiency virus
SD: Standard deviation
SPSS: Statistical package for social science
TB: Tuberculosis
WHO: World Health Organization
